# Expression of the cancer stem cell marker SSEA1 is associated with poor survival in metastatic high-grade serous carcinoma

**DOI:** 10.1007/s00428-020-02850-4

**Published:** 2020-05-29

**Authors:** Ben Davidson, Arild Holth, Hiep Phuc Dong

**Affiliations:** 1 Department of Pathology, Oslo University Hospital, Norwegian Radium Hospital, N-0310 Oslo, Norway; 2 Faculty of Medicine, Institute of Clinical Medicine, University of Oslo, N-0316 Oslo, Norway

**Keywords:** Cancer stem cells, SSEA1, Immunohistochemistry, Flow cytometry, High-grade serous carcinoma, Effusion

## Abstract

The objective of the present study was to perform a quantitative analysis of cancer stem cell (CSC) marker expression in ovarian carcinoma effusions. The clinical role of SSEA1 in metastatic high-grade serous carcinoma (HGSC) was additionally analyzed. CD133, Nanog, SOX2, Oct3/4, SSEA1, and SSEA4 protein expressions were quantitatively analyzed using flow cytometry (FCM) in 24 effusions. SSEA1 expression by immunohistochemistry was analyzed in 384 HGSC effusions. Highly variable expression of CSC markers by FCM was observed, ranging from 0 to 78% of Ber-EP4-positive cells in the case of CD133, with the largest number of negative specimens seen for SSEA4. SSEA1 expression by immunohistochemistry was found in HGSC cells in 336/384 (89%) effusions, most commonly focally (< 5% of cells). SSEA1 was overexpressed in post-chemotherapy disease recurrence specimens compared with chemo-naïve HGSC effusions tapped at diagnosis (*p* = 0.029). In univariate survival analysis, higher SSEA1 expression was significantly associated with poor overall survival (*p* = 0.047) and progression-free survival (*p* = 0.018), though it failed to retain its prognostic role in Cox multivariate survival analysis in which it was analyzed with clinical parameters (*p* = 0.059 and *p* = 0.111 for overall and progression-free survival, respectively). In conclusion, CSC markers are variably expressed in ovarian carcinoma effusions. SSEA1 expression is associated with disease progression and poor survival in metastatic HGSC. Silencing this molecule may have therapeutic relevance in this cancer.

## Introduction

Ovarian cancer, consisting predominantly of ovarian carcinoma (OC), constitutes the 8th most common cancer and the 8th most common cause of cancer death in women globally, with 295,414 new diagnoses and 184,799 deaths in 2018 [1]. Despite improvement in survival in recent years, due to optimized surgery and chemotherapy protocols, as well as targeted therapy, 5-year survival is only 45%. Furthermore, this figure is true for all histological types combined, and outcome is still worse for patients diagnosed with high-grade serous carcinoma (HGSC), the most common and aggressive type of OC, in which diagnosis is often at advanced stage (FIGO stages III–IV) and death-of-disease occurs within 5 years in the majority of patients [2]. HGSC has its origin most frequently in the fallopian tube, and metastasizes widely within the peritoneal cavity, forming both solid lesions and malignant ascites. Involvement of the pleural space is the most common manifestation of stage IV disease.

Molecules that have been reported to be cancer stem cell (CSC) markers in OC include the surface proteins CD24, CD44, CD117, and CD133, and the intracellular cytoplasmic and/or nuclear proteins aldehyde dehydrogenase isoform 1A1 (ALDH1A1), OCT4, Nanog, SOX2, Notch-1, and nestin, as well as the detection of a side population by flow cytometry (FCM) [reviewed in 3, 4]. The majority of these markers have been shown to be expressed in OC cells in effusions, an anatomic niche characterized by anoikis resistance and chemoresistance [reviewed in 5–7].

Our group previously reported on expression of CD24 and nestin in serous OC effusions, though expression of both CSC markers was unrelated to chemotherapy response or survival [8, 9]. Recently, we identified SOX2 and SOX9 as markers of poor chemotherapy response and shorter survival in analysis of HGSC cells in effusions [10].

The objective of the present study was to quantitatively analyze the expression of CD133, previously not analyzed in our cohort, in OC effusions, and to assess whether this protein was co-expressed with other CSC markers, which was not feasible in our earlier studies in which immunohistochemistry (IHC) and quantitative RT-PCR were applied. We additionally studied the clinical role of the CSC marker SSEA1 in a large cohort of patients with HGSC effusions, the majority diagnosed at FIGO stages III–IV.

## Material and methods

### Patients and specimens

#### Flow cytometry cohort

Effusions consisted of 24 specimens (21 peritoneal, 3 pleural) submitted to the Department of Pathology at the Norwegian Radium Hospital during the period of 2005 to 2014. Effusions were centrifuged immediately after tapping, and cell pellets were frozen at − 70 °C in equal amounts of RPMI 1640 medium (GIBCO-Invitrogen, Carlsbad, CA) containing 50% fetal calf serum (PAA Laboratories GmbH, Pasching, Austria) and 20% dimethylsulfoxide (Merck KGaA, Darmstadt, Germany). Surgical specimens from the 24 patients were diagnosed by an experienced gyn-pathologist (BD) as HGSC (*n* = 17), low-grade serous carcinoma (LGSC; *n* = 4), endometrioid carcinoma (EC; *n* = 1), clear cell carcinoma (CCC; *n* = 1), and carcinosarcoma (CS; *n* = 1). Effusion specimens were diagnosed as adenocarcinoma/CS metastases by an experienced cytopathologist (BD) based on morphology in smears and cell blocks prepared using the thrombin clot protocol and IHC, based on established guidelines [5].

#### IHC cohort

HGSC effusions (*n* = 384; 327 peritoneal, 57 pleural) from 384 patients were submitted to the Department of Pathology at the Norwegian Radium Hospital during the period of 1998 to 2015. Effusions were centrifuged immediately after tapping, and cell pellets were used for preparation of cell blocks using the thrombin clot protocol. Clinicopathologic data are detailed in Table 1.

**Table 1:** Clinicopathologic parameters of the HGSC effusion cohort (384 patients)

Parameter		Distribution
Age (mean)		23–88 years (63)
FIGO stage		
I		3
II		7
III		223
IV		148
NA		3
Residual disease		
Primary debulking surgery (*n* = 204)	0 cm	31
	≤ 1 cm	83
	> 1 cm	90
Interval debulking surgery (*n* = 103)	0 cm	27
	≤ 1 cm	46
	> 1 cm	30
NA		77
CA 125 at diagnosis (range; median)		10–62,400 (1237)^a^
Chemoresponse after primary treatment		
CR		177
PR		95
SD		28
PD		39
NA^b^		45

*NA*, not available; *CR*, complete response; *PR*, partial response; *SD*, stable disease; *PD*, progressive disease

^a^Available for 304 patients

^b^Not available (missing data or disease response after chemotherapy could not be evaluated because of normalized CA 125 after primary surgery or missing CA 125 information and no residual tumor)

Informed consent was obtained according to national and institutional guidelines. Study approval was given by the Regional Committee for Medical Research Ethics in Norway.

### FCM

FCM (flow cytometry) was undertaken using the FACSCalibur flow cytometer (Becton-Dickinson, San Jose, CA) equipped with a 15-mW Argon-ion laser (488 nm) and 12-mW red diode laser (635 nm). Filter configurations were fluorescein isothiocyanate (FITC, FL1, BP 530/30 nm), phycoerythrin (PE, FL2, BP 585/42 nm), peridinin chlorophyll protein (PerCP, FL3, LP 670 nm), and allophycocyanin (APC, FL4, BP 661/16 nm). Forward light scatter channel (FSC) and side angle light scatter channel (SSC) parameters were defined in linear amplification mode, and all fluorescence parameters (FL1, FL2, FL3, and FL4) were defined in logarithmic amplification mode. For each measurement, data from at least 100,000 events were collected.

Control of instrument performance and time delay calibration were performed using the FACSComp software version 4.1, Calibrite™ 3 beads, and Calibrite™ APC beads (Becton-Dickinson) for four-color flow cytometer setup. Threshold was based on FSC as a primary parameter and compensation settings were determined as previously described [11].

The OVCAR-3 and OVCAR-8 HGSC cell lines were tested for CSC marker expression, and the former was chosen as positive control.

#### Sample preparation

Frozen material was thawed and 10-ml RPMI 1640 with 10% FCS was added. After centrifugation for 5 min at 1200 rpm, the supernatant was decanted and 2-ml incubation buffer (Cell Signaling Technology, Danvers, MA) was added in each sample. Cell suspensions were mixed gently with a pipette, filtered through a 70-μm BD Falcon™ cell strainer, and centrifuged for 5 min at 1200 rpm. Subsequently, 2-ml incubation buffer was added and cells were blocked for non-specific binding in incubation buffer for 10 min at room temperature, followed by division of 100 μl of cell suspension (2 × 10^6^ cells) into separate tubes for surface and intracellular staining. The antibodies and combinations employed are detailed in Tables 2 and 3.

**Table 2:** Antibodies employed in FCM analysis

Antibody	Source	Clone	Cat. no.
CD133-PE-Cy5.5	MACS Miltenyi Biotec (Bergisch Gladbach, Germany)	AC133	130-090-422
SSEA1-PE	R&D systems, Inc. (Minneapolis, MN)	MC-480	FAB2155P
SSEA4-PE	BD Biosciences Pharmingen (San Jose, CA)	MC813-70	560128
Nanog-PE	BD Biosciences Pharmingen	N31-355	560873
SOX2-PE	BD Biosciences Pharmingen	O30-678	562195
Oct3/4A-PE	BD Biosciences Pharmingen	O50-808	561556
CD45-APC	BD Biosciences Pharmingen	2D1	340910
Ber-EP4-FITC	Dako (Glostrup, Denmark)	Ber-EP4	F0860
IgG1-FITC	Dako	DAK-GO1	X0964
Goat Anti-Mouse IgG-PE-Cy5.5	Invitogen (Carlsbad, CA)		M32018
Fixable Viability Dye eFlour 660	eBioscience (San Diego, CA)		65-0864-14
IgG3-PE	BD Biosciences Pharmingen		559926
IgG1-PE	BD Biosciences Pharmingen	MOPC-21	554680
IgM- PE	R&D systems, Inc.		IC015P

*FITC*, fluorescein isothiocyanate; *PE*, phycoerythrin; *PeCy5.5*, phycoerythrin cyanine5.5; *APC*, allophycocyanin; *FMO*, Fluorescence Minus One

**Table 3:** Combinations employed for FCM

Tube no.	Test	FITC	PE	PeCy5.5	APC/efluor660
1	Control of overall background staining	Isotype	Isotype	Isotype	Isotype
2	Control of secondary antibody	Ber-EP4 5 μl	Isotype	Secondary goat-anti-mouse PE-cy5.5 2.5 μl	CD45/eFluor660 5 μl/1 μl
3	Ber-EP4 FMO	FMO	SSEA1 10 μl	CD133 10 μl	CD45/eFluor660 5 μl/1 μl
4	SSEA1/SSEA4 FMO	Ber-EP4 5 μl	FMO	CD133 10 μl	CD45/eFluor660 5 μl/1 μl
5	SSEA1/CD133 (in carcinoma cells)	Ber-EP4 5 μl	SSEA1 10 μl	CD133 10 μl	CD45/eFluor660 5 μl/1 μl
6	SSEA4/CD133 (in carcinoma cells)	Ber-EP4 5 μl	SSEA4 20 μl	CD133 10 μl	CD45/eFluor660 5 μl/1 μl
7	FMO cy	Ber-EP4 5 μl	FMO Cy Isotype IgG1-5 μl	CD133 10 μl	CD45/eFluor660 5 μl/1 μl
8	Nanog/CD133 (in carcinoma cells)	Ber-EP4 5 μl	Cy Nanog 20 μl	CD133 10 μl	CD45/eFluor660 5 μl/1 μl
9	SOX2/CD133 (in carcinoma cells)	Ber-EP4 5 μl	Cy SOX2 5 μl	CD133 10 μl	CD45/eFluor660 5 μl/1 μl
10	Oct3/4A/CD133 (in carcinoma cells)	Ber-EP4 5 μl	Cy Oct3/4A 5 μl	CD133 10 μl	CD45/eFluor660 5 μl/1 μl

*FITC*, fluorescein isothiocyanate; *PE*, phycoerythrin; *PeCy5.5*, phycoerythrin cyanine5.5; *APC*, allophycocyanin; *FMO*, Fluorescence Minus One

#### Surface staining

Primary monoclonal non-conjugated mouse anti-human antibody CD133 for surface staining was added to respective tubes and cells were vortexed and incubated at room temperature for 30 min. Each tube was washed twice with 2-ml incubation buffer followed by centrifugation for 5 min at 1200 rpm and decanting of the supernatant. Secondary PeCy5.5 conjugated goat anti-mouse antibody was added to respective tubes and cells were vortexed and incubated for 30 min at room temperature. Each tube was washed twice with 2-ml incubation buffer followed by centrifugation for 5 min at 1200 rpm and decanting of the supernatant. Primary monoclonal direct-conjugated mouse anti-human antibodies (isotype mouse IgG1, Ber-EP4, SSEA-1, SSEA-4, CD45, and Fixable Viability Dye eFlour™ 660) for surface staining were added to their respective tubes. Cells were vortexed and incubated in the dark for 30 min at room temperature. At the end of incubation, the washing step was repeated twice with 2-ml incubation buffer following addition of 200-μl FacsFlow sheath fluid (Becton-Dickinson) to each tube. Samples were then kept on ice until analysis.

#### Intracellular staining

A volume of 100 μl of medium A (FIX & PERM reagents, Caltag Laboratories, Invitrogen, Carlsbad, CA) was added to the relevant tubes and incubated for 15 min at room temperature in the dark. Cells were then washed twice with 2-ml phosphate buffer saline (PBS) followed by centrifugation for 5 min at 1200 rpm and decanting of the supernatant. A volume of 100 μl of medium B (FIX & PERM reagents, Caltag Laboratories) was added to the relevant tubes and incubated at room temperature for 15 min in the dark. Cells were then washed twice with 2-ml PBS, followed by addition of the primary antibodies (Nanog, SOX2 and Oct3/4A) for intracellular staining in the relevant tubes and incubation at room temperature for 30 min in the dark. Cells were washed twice with 2-ml incubation buffer and centrifuged for 5 min at 1200 rpm. At the end of incubation, the washing step was repeated twice with 2-ml incubation buffer following addition of 200-μl FacsFlow sheath fluid (Becton-Dickinson) to each tube. Samples were then kept on ice until analysis.

#### Evaluation of FCM immunophenotyping

Analysis of FCM results was undertaken in a standardized way by using the FlowJo version 9.7.6 analysis software (Tree Star Inc., Ashland, OR). A gating procedure was generated by analyzing SSC versus CD45-APC fluorescence/eFluor™ 660 and a region was drawn around clear-cut populations having negative CD45-APC fluorescence/eFluor™ 660. Cells in this region were again viewed by generating a cytogram combining SSC versus FSC, and a gating procedure was used in order to exclude cell debris, by including only cells with relatively high SSC and FSC values. Quadrant cursors were set by using isotypic negative controls and the Fluorescence Minus One (FMO) control. Quadrant settings were chosen so that in negative controls, 99% of the cells were localized in the left lower quadrant. The percentage of carcinoma cells expressing CD133, SSEA1, SSAE4, Nanog, SOX2, and Oct 3/4A was scored. Expression in < 1% of cells was scored as negative.

### IHC

Formalin-fixed, paraffin-embedded sections from 384 HGSC effusions were analyzed for SSEA1 protein expression using the Dako EnVision Flex + System (K8012; Dako, Glostrup, Denmark). The SSEA1 antibody was a mouse monoclonal antibody purchased from R&D Biosystems (cat # MAB2155, clone MC-480; Minneapolis, MN), applied at a 1:200 dilution following antigen retrieval in citrate buffer (pH 6.0).

Following deparaffinization, sections were treated with EnVision™ Flex + mouse linker (15 min) and EnVision™ Flex/HRP enzyme (30 min) and stained for 10 min with 3′3-diaminobenzidine tetrahydrochloride (DAB), counterstained with hematoxylin, dehydrated, and mounted in Richard-Allan Scientific Cyto seal XYL (Thermo Fisher Scientific, Waltham, MA). Positive control consisted of normal testis. In negative controls, the primary antibody was replaced with isotype-specific mouse myeloma protein diluted to the same concentration as the primary antibody.

#### IHC scoring

Membrane staining was interpreted as positive. Staining was scored by an experienced cytopathologist (BD), using a 0–4 scale as follows: 0 = no staining, 1 = 1–5%, 2 = 6–25%, 3 = 26–75%, 4 = 76–100% of tumor cells.

### Statistical analysis

Statistical analysis was performed by applying the SPSS-PC package (Version 26). Probability of < 0.05 was considered statistically significant. The Mann-Whitney *U* test or the Kruskal-Wallis *H* test was applied to analysis of the association between SSEA1 protein expression by IHC and clinicopathologic parameters (for 2-tier or 3-tier analyses, respectively). For this analysis, clinicopathologic parameters were grouped as follows: age: ≤ 60 vs. > 60 years; effusion site: peritoneal vs. pleural; FIGO stage: III vs. IV; chemotherapy status: pre- vs. post-chemotherapy specimens; residual disease (RD) volume: 0 cm vs. ≤ 1 cm vs. > 1 cm; response to chemotherapy: complete response vs. partial response/stable disease/progressive disease. Progression-free survival (PFS) and overall survival (OS) were calculated from the date of the last chemotherapy treatment/diagnosis to the date of recurrence/death or last follow-up, respectively. Univariate survival analyses of PFS and OS were executed using the Kaplan-Meier method and log-rank test. Multivariate survival analysis was executed using the Cox regression model. Platinum resistance was defined as PFS ≤ 6 months according to guidelines published by the Gynecologic Oncology Group (GOG) and progressive disease or recurrence was evaluated by the response evaluation criteria in solid tumors (RECIST) criteria.

## Results

### CSC markers are differentially expressed in OC effusions

**Fig. 1:**
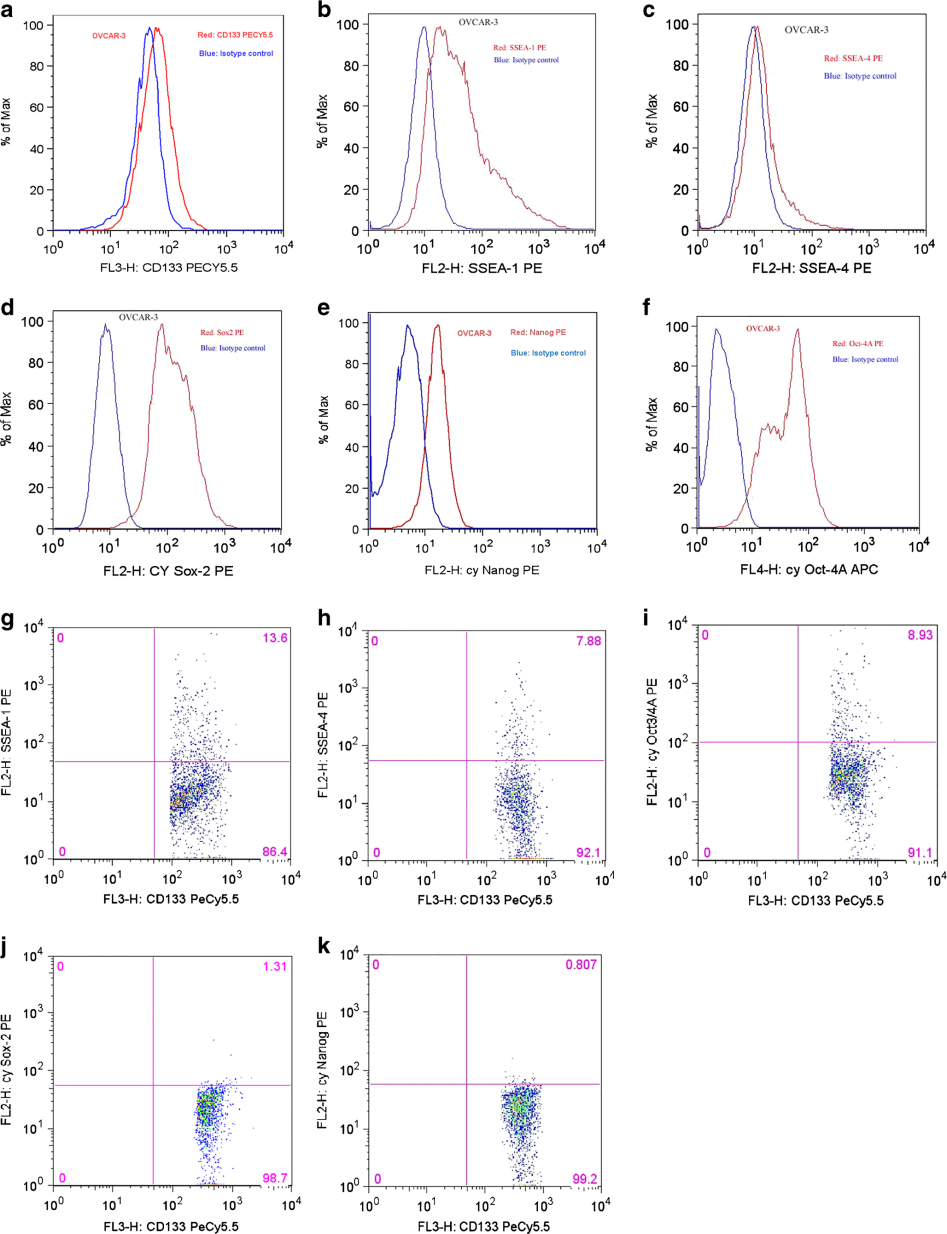
Flow cytometry. **a**–**f** Expression of CSC markers in the control HGSC cell line OVCAR-3. **g**–**k** Co-expression of CD133 with SSEA1 (**g**), SSEA4 (**h**), Oct3/4 (**i**), SOX2 (**j**), and Nanog (**k**). All proteins except SSEA1 have low co-expression levels (< 10% of cells)

The expression of CSC markers in OC cells was analyzed by gating on Ber-EP4-positive, CD45-negative cells. Highly variable expression of all markers was observed (Table 4), though median expression was < 5% of cells for all markers, with highest number of negative specimens observed for SSEA4. Analysis of co-expression of CD33, previously reported to be a robust CSC marker in OC [3, 4], with the 5 remaining CSC markers showed highest level of co-expression with SSEA1, reaching 24% in a case of CCC, with values < 10% for the other 4 markers (data not shown; representative case illustrated in Fig. 1).

**Table 4:** Expression of CSC markers in carcinoma cells by FCM

Case	Diagnosis	CD133	SSEA1	SSEA4	Nanog	SOX2	Oct3/4
1	HGSC	8	11	0	3	4	0
2	HGSC	0	13	3	13	6	4
3	HGSC	0	11	0	5	8	8
4	HGSC	5	25	0	3	15	0
5	LGSC	35	28	19	0	1	23
6	HGSC	8	21	0	0	2	2
7	HGSC	3	6	0	11	13	3
8	EC	10	1	0	0	1	0
9	HGSC	7	0	0	22	11	50
10	HGSC	0	0	0	12	42	1
11	LGSC	1	1	0	13	15	1
12	LGSC	7	1	4	2	0	1
13	HGSC	22	24	1	3	3	20
14	HGSC	13	9	2	2	2	8
15	HGSC	53	1	1	2	3	7
16	HGSC	5	0	0	2	2	1
17	HGSC	2	1	1	6	21	13
18	LGSC	2	0	0	4	2	3
19	HGSC	3	11	0	0	0	0
20	CS	0	0	10	2	2	2
21	HGSC	2	0	0	2	1	2
22	HGSC	0	0	0	6	8	29
23	HGSC	3	0	0	11	6	12
24	CCC	78	22	0	3	3	1

*HGSC*, high-grade serous carcinoma; *LGSC*, low-grade serous carcinoma; *EC*, endometrioid carcinoma; *CCC*, clear cell carcinoma; *CS*, carcinosarcoma

**Fig. 2:**
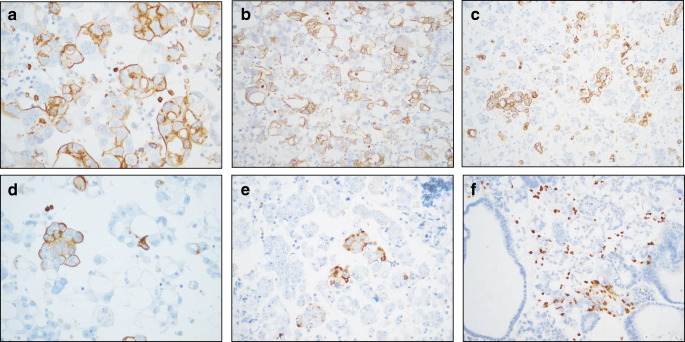
Immunohistochemistry. SSEA1 protein expression in 6 HGSC effusions. **a**–**c** Expression in the majority of tumor cells; **d**–**e** Focal expression; **f** SSEA1-negative tumor, with staining in lymphocytes, known to express this molecule (see discussion)

As Nanog, SOX2, and Oct3/4 were previously studied in our cohort [10], and given the fact that SSEA4 expression by FCM was low, we expanded the study with respect to SSEA1, analyzing a large series of HGSC effusions. In agreement with the FCM data, SSEA1 expression by IHC was found in HGSC cells in 338/386 (88%) effusions (Fig. 2). Staining extent was as follows: 0: 48 effusions; 1: 206 effusions; 2: 65 effusions; 3: 53 effusions; 4: 12 effusions. SSEA1 was significantly overexpressed in post-chemotherapy effusions compared with pre-chemotherapy specimens tapped at diagnosis (*p* = 0.029), though its levels were not significantly related to other clinicopathologic parameters (*p* > 0.05; data not shown).

Data regarding OS were available for all 384 patients, whereas 372 had data regarding PFS. The follow-up period ranged from 1 to 179 months (mean = 37 months, median = 29 months). PFS ranged from 0 to 148 months (mean = 11 months, median = 7 months). At the last follow-up, 348 patients were dead of disease, 23 were alive with disease, and 5 were with no evidence of disease. Four patients died of complications and 4 patients were lost to follow-up.

**Fig. 3:**
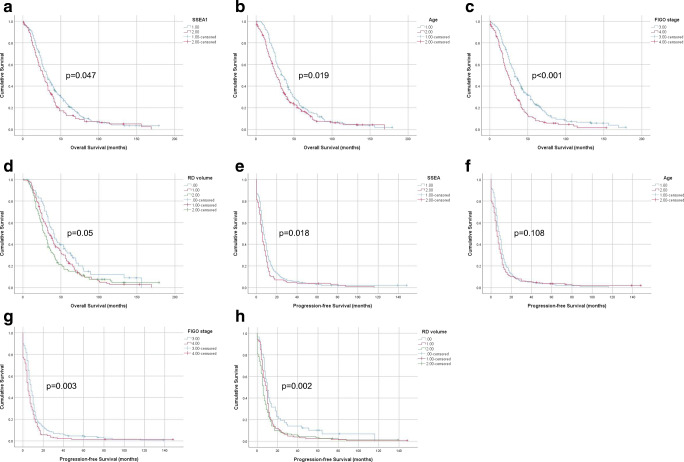
Survival. **a** Kaplan-Meier survival curve showing the association between SSEA1 protein expression and overall survival (OS) for 384 HGSC patients. Patients with effusions with high (> 5%) SSEA1 expression (*n* = 130; red line) had mean OS of 36.6 months compared with 42.9 months for patients with effusions having low (≤ 5%) SSEA1 expression (*n* = 254, blue line; *p* = 0.047). **b** Kaplan-Meier survival curve showing the association between patient age and OS for 384 HGSC patients. Older (> 60 years) patients (*n* = 224; red line) had mean OS of 37.5 months compared with 45.1 months for younger (≤ 60 years) patients (*n* = 160, blue line; *p* = 0.019). **c** Kaplan-Meier survival curve showing the association between FIGO stage and OS for 371 HGSC patients with advanced-stage disease. Patients diagnosed with stage IV disease (*n* = 148; red line) had mean OS of 30.8 months compared with 45.8 months for patients with stage III disease (*n* = 223, blue line; *p* < 0.001). **d** Kaplan-Meier survival curve showing the association between residual disease (RD) volume and OS for 306 patients with debulking data. Patients debulked to no macroscopic disease (*n* = 58; blue line) had mean OS of 54.3 months compared with 44.1 and 40 months for patients debulked to 1 cm (*n* = 128, red line) and ≥ 2 cm (*n* = 120, green line), respectively (*p* = 0.05). **e** Kaplan-Meier survival curve showing the association between SSEA1 protein expression and progression-free survival (PFS) for 372 HGSC patients with PFS data. Patients with effusions with high (> 5%) SSEA1 expression (*n* = 127; red line) had mean PFS of 9.9 months compared with 13 months for patients with effusions having low (≤ 5%) SSEA1 expression (*n* = 245, blue line; *p* = 0.018). **f** Kaplan-Meier survival curve showing the association between patient age and PFS for 372 HGSC patients with PFS data. Older (> 60 years) patients (*n* = 214; red line) had mean PFS of 11.6 months compared with 12.1 months for younger (≤ 60 years) patients (*n* = 158, blue line; *p* = 0.108). **g** Kaplan-Meier survival curve showing the association between FIGO stage and PFS for 360 HGSC patients with advanced-stage disease and PFS data. Patients diagnosed with stage IV disease (*n* = 143; red line) had mean PFS of 8.9 months compared with 13 months for patients with stage III disease (*n* = 217, blue line; *p* = 0.003). **h** Kaplan-Meier survival curve showing the association between residual disease (RD) volume and PFS for 303 patients with debulking and PFS data. Patients debulked to no macroscopic disease (*n* = 57; blue line) had mean PFS of 20.9 months compared with 12 and 10.7 months for patients debulked to 1 cm (*n* = 126, red line) and ≥ 2 cm (*n* = 120, green line), respectively (*p* = 0.002)

In view of the large number of cases with negative or focal (≤ 5%) SSEA1 expression, survival analysis compared tumors with focal/negative expression with those expressing this protein in > 5% of carcinoma cells. In univariate analysis of OS, higher SSEA1 expression was significantly associated with shorter survival (*p* = 0.047; Fig. 3a). Among clinical parameters, older age (*p* = 0.019; Fig. 3b) and FIGO stage IV (*p* < 0.001; Fig. 3c) were significantly related to shorter OS, with marginal significance for RD volume (*p* = 0.05; Fig. 3d). In Cox multivariate survival analysis, in which these 4 parameters were entered, only FIGO stage emerged as independent prognosticator, although a trend was observed for SSEA1 (SSEA1: *p* = 0.059; age: *p* = 0.739; FIGO stage: *p* < 0.001; RD volume: *p* = 0.126).

Higher SSEA1 expression was additionally significantly associated with poorer outcome in univariate analysis of PFS (*p* = 0.018; Fig. 3e). Among clinical parameters, age was not significantly related to PFS (*p* = 0.108; Fig. 3f), whereas FIGO stage IV (*p* = 0.003; Fig. 3g) and larger RD volume (*p* = 0.002; Fig. 3h) were strongly related to shorter PFS. In Cox multivariate survival analysis, in which these 4 parameters were entered, only RD volume emerged as independent prognosticator, with a trend for FIGO stage (SSEA1: *p* = 0.111; age: *p* = 0.966; FIGO stage: *p* = 0.055; RD volume: *p* = 0.006).

## Discussion

HGSC, particularly when diagnosed at advanced stage, is characterized by a tendency to recur, even following optimal debulking and complete response to chemotherapy at diagnosis, eventually leading to death of the majority of patients. Recurrence is mediated by cells that survived chemotherapy and may thus have CSC phenotype.

The first objective of the present study was to assess the expression by FCM of 6 CSC markers in Ber-EP4-positive, CD45-negative carcinoma cells. This analysis showed variable expression of all 6 markers, with only few cases showing diffuse expression (defined as > 25% in our studies using IHC) of any given marker. Stage-specific embryonic antigen 4 (SSEA4) was the least frequently expressed protein. Although the number of specimens analyzed is too small to draw conclusions regarding differences among different histological types of ovarian carcinoma, it is noteworthy that the highest CD133 expressor (78% of cells) was a CCC, a tumor known for its chemoresistance.

Data with respect to SOX2 and Oct3/4 are in agreement with our recent observation that expression of these markers by IHC in HGSC effusions is variable, and often limited to < 50% of cells in HGSC effusions. In contrast, Nanog expression, though predominantly limited to < 10% of tumor cells, was higher than in the former study, where this protein was absent from tumor cells and was found mostly in secreted exosomes [10]. The reason for this may be the increased sensitivity of FCM, a method analyzing fresh-frozen viable cells, compared with IHC, although other technical differences or factors related to the different cohorts cannot be excluded.

Two proteins which have not been previously studied in our cohort were SSEA1 and SSEA4. SSEA antibodies react with specific glycosphingolipids (GSL), a family of molecules localized in the outer leaflet of the plasma membrane, which currently consists of more than 1000 members and are divided into 4 groups based on their core structure—the globo, lacto, neolacto, and ganglio series. SSEA1 antibodies recognize the neolacto series epitope Le^x^ and SSEA4 antibodies bind the globo series epitope monosialyl-GB5. Changes in glycan phenotypes are observed during embryogenesis, and SSEAs are considered markers of embryonic stem cells [12, 13].

Cells expressing SSEA4, considered to be pluripotent, were identified in normal ovaries [14], and SSEA4-positive cells are found in HGSC [15]. SSEA4 expression was additionally reported to be marker of chemoresistance in breast carcinoma [16]. SSEA1 is a leukocyte marker which has additionally been extensively studied, under its acronyms CD15/Leu-M1, as a diagnostic marker for a variety of carcinomas. In the context of ovarian carcinoma, it has been shown to be a highly specific marker for serous carcinoma in the differential diagnosis from malignant mesothelioma [17], though it has been less frequently used than more sensitive (though less specific) markers such as Ber-EP4/MOC31 in recent years. As with SSEA4, expression of Le^x^ entities was reported to be associated with chemoresistance [18].

To the best of our knowledge, no data regarding the clinical relevance of SSEA1 or SSEA4 in HGSC effusions is available to date. As SSEA4 expression by FCM was very limited, we focused on SSEA1 expression in an expanded series of well-characterized and clinically annotated HGSC effusions.

We observed distinct, albeit frequently focal, SSEA1 expression in HGSC cells, a pattern resembling staining for other carbohydrate markers that we use in the routine diagnosis of serous effusions, such as B72.3 [19]. Although we did not study malignant mesotheliomas, SSEA1 expression was uniformly absent in reactive mesothelial cells, well in agreement with the excellent performance of this marker in differentiating these entities. SSEA1 was significantly overexpressed in post-chemotherapy effusions compared with pre-chemotherapy specimens tapped at diagnosis. Although these groups did not consist of patient-matched pre- and post-chemotherapy specimens, this difference may suggest selection of chemotherapy-resistant SSEA1-expressing tumor cells along disease progression. Although SSEA1 expression was unrelated to chemotherapy response, it was significantly associated with shorter PFS and OS, suggesting it may be a marker of more aggressive clinical course in metastatic HGSC. It was not, however, an independent prognosticator in Cox multivariate analysis, a fact which reflects the inherent power of clinical parameters, such as FIGO stage and RD volume, in this malignancy.

There are several limitations to our study. The FCM analysis was performed on a small number of cases, and histotypes other than HGSC are represented by single or very few cases. Both the FCM and IHC cohorts are retrospective, the latter with a considerable time span during which several treatment aspects related to OC, e.g., optimal RD volume and use of anti-angiogenic therapy, have been modified. Finally, the inherent limitations of non-quantitative methodology such as IHC, in particular subjective scoring, need to be taken into account. Larger studies of histotypes other than HGSC, as well as prospective studies of the latter histologic type, are therefore necessary in order to confirm or refute the findings in the present study.

In conclusion, CSC markers are variably expressed in OC effusions, predominantly limited to small cell populations. SSEA1 is more frequently co-localized with CD133 than other CSC markers, is overexpressed following chemotherapy, and is a marker of shorter survival. Its association and clinical role in tumors characterized by chemoresistance, such as CCC, merit further research.
